# Niemann–Pick type C2-mediated cholesterol sequestration induces capacitation-like sperm remodeling during reproductive tract transit in the blue swimming crab (Portunus pelagicus)

**DOI:** 10.14202/vetworld.2026.3651-3667

**Published:** 2026-08-27

**Authors:** Wattana Weerachatyanukul, Thanyaporn Senarai, Thitiporn Khongkha, Orawan Thongsum, Supawich Boonkua, Jirawat Saetan, Piyaporn Surinlert, Somluk Asuvapongpatana

**Affiliations:** 1 Department of Anatomy, Faculty of Science, Mahidol University, Bangkok 10400, Thailand.; 2 Department of Anatomy, Faculty of Medicine, Khon Kaen University, Khon Kaen 40002, Thailand.; 3 Chulabhorn International College of Medicine, Thammasat University, Pathum Thani 12120, Thailand.; 4 Division of Health and Applied Sciences, Faculty of Science, Prince of Songkla University, Songkhla 90110, Thailand.

**Keywords:** Brachyuran crab, cholesterol sequestration, external fertilization, Niemann–Pick type C2, Portunus pelagicus, protein tyrosine phosphorylation, sperm capacitation-like process, sperm remodeling

## Abstract

**Background and Aim:**

Cholesterol (CHO) sequestration from the sperm plasma membrane is a fundamental event during mammalian sperm capacitation, but the molecular mechanisms underlying capacitation-like sperm activation in brachyuran crabs remain unknown. Although Niemann–Pick type C2 (NPC2), a high-affinity CHO-binding protein, has been implicated in sperm maturation in penaeid shrimp, its occurrence and physiological role in brachyuran species have not been investigated. This study characterized the *Portunus pelagicus* NPC2 (*PpNPC2*) gene, determined its localization in the male reproductive tract, and evaluated its role in capacitation-like sperm remodeling during reproductive tract transit in CHO-dependent capacitation.

**Materials and Methods:**

The *PpNPC2* sequence was identified and analyzed using comparative molecular and phylogenetic approaches. Localization of PpNPC2 in the testis and spermatic duct was examined by immunohistochemistry. Recombinant PpNPC2 protein was produced in *Escherichia coli* and functionally evaluated using isolated testicular sperm. Membrane CHO content was quantified by high-performance thin-layer chromatography and Filipin III fluorescence staining, whereas sperm morphology and protein tyrosine phosphorylation were assessed using light microscopy, immunofluorescence, and Western blotting.

**Results:**

*PpNPC2* encoded a conserved NPC2 protein that clustered phylogenetically with crustacean NPC2 homologs and was predominantly localized in epithelial cells of the testis and spermatic duct. During sperm transit, membrane CHO decreased by approximately 32%, accompanied by progressive transformation of round testicular sperm into characteristic snowman-like spermatic duct sperm. Recombinant PpNPC2 reproduced these physiological changes by promoting CHO sequestration, inducing time- and concentration-dependent morphological remodeling, and significantly increasing protein tyrosine phosphorylation by up to 2.33-fold (p < 0.01). These findings indicate that PpNPC2 functions as a CHO-sequestering protein that initiates capacitation-like biochemical and structural remodeling during sperm maturation.

**Conclusion:**

This study provides the first molecular and functional characterization of NPC2 in a brachyuran crab and demonstrates that NPC2-mediated CHO sequestration is a conserved mechanism driving capacitation-like sperm activation during reproductive tract transit. These findings expand current understanding of reproductive biology in marine crustaceans and provide a molecular foundation for future studies on sperm preservation, assisted reproduction, and crustacean aquaculture.

## INTRODUCTION

In mammalian sperm physiology, the cholesterol (CHO)-to-phospholipid ratio is an important determinant of sperm capacitation and fertilization potential [1]. During capacitation, sperm membranes undergo lipid remodeling, lateral redistribution, and increased membrane fluidity, partly because of CHO efflux into the extracellular environment [2]. These biochemical changes subsequently promote the formation of sterol-rich microdomains, known as lipid rafts, which serve as platforms for receptor activation and signal transduction pathways essential for fertilization [3, 4]. Capacitation also involves activation of intracellular signaling pathways mediated by cyclic adenosine monophosphate (cAMP) and protein kinase A (PKA), resulting in increased protein tyrosine phosphorylation, a widely recognized biomarker of sperm activation [5]. In addition to CHO efflux, recent studies have demonstrated that capacitation is regulated by complex interactions among membrane ion channels, intracellular pH, and dynamic lipid redistribution. These findings indicate that sperm activation is an integrated biochemical, biophysical, and signaling process rather than solely a lipid-efflux event [6–8].

Serum albumin, a major CHO acceptor in the female reproductive tract, is commonly used during *in vitro* fertilization to induce sperm capacitation [9, 10]. Niemann–Pick type C2 (NPC2), another well-recognized high-affinity CHO-binding protein, is involved in sperm maturation and membrane lipid modulation [11–13]. In crustaceans, NPC2 orthologs have been identified in shrimp reproductive tissues, where they are implicated in the removal of CHO from sperm membranes and subsequent physiological activation during sperm transit from the testis to the spermatic duct [14–16]. Comparative structural modeling and ligand-binding analyses have demonstrated evolutionary conservation of NPC2, particularly its sterol-binding domains, across vertebrates and invertebrates, supporting its role in sperm membrane homeostasis [17–19]. Furthermore, advances in lipidomics, sterol imaging, and mass spectrometry have enabled more precise characterization of lipid remodeling in the sperm membrane during sperm activation and fertilization [20, 21]. Previous studies identified *Macrobrachium rosenbergii* NPC2 (MrNPC2) in epithelial cells and luminal fluid of the vas deferens and demonstrated its contribution to sperm membrane lipid homeostasis and activation [14–16]. These findings suggest that NPC2-mediated CHO sequestration in crustacean sperm resembles mechanisms involved in mammalian sperm capacitation [1, 2].

Although NPC2-mediated sperm membrane remodeling has been investigated in penaeid prawns, its molecular characteristics and physiological functions in brachyuran crabs remain unexplored. This distinction is important because penaeid sperm have a relatively simple structure comprising a nucleus and an acrosomal vesicle with limited motility [14], whereas brachyuran sperm are aflagellated and non-motile and possess highly specialized acrosomal structures. During activation, the prominent acrosomal filaments of brachyuran sperm produce a characteristic snowman-like morphology [22]. Therefore, CHO-dependent membrane and acrosomal remodeling in brachyuran sperm may represent an evolutionarily distinct reproductive strategy adapted to external fertilization in marine environments. However, the proteins that regulate this process during sperm transit through the male reproductive tract have not been identified.

This study aimed to characterize the full-length *Portunus pelagicus NPC2* (*PpNPC2*) gene and its predicted molecular structure, determine the localization of PpNPC2 protein in the male reproductive tract, and investigate its role in CHO sequestration and capacitation-like sperm remodeling. The effects of recombinant PpNPC2 on sperm morphology and protein tyrosine phosphorylation were also evaluated to determine whether this protein contributes to the acquisition of fertilization readiness during sperm transit through the spermatic duct.

## MATERIALS AND METHODS

### Ethical approval

All experimental procedures involving animals were reviewed and approved by the Animal Care and Use Committee (IACUC Review), Faculty of Science, Mahidol University, Bangkok, Thailand (protocol numbers MUSC63-018-526 and MUSC67-063-768). All experimental procedures were conducted in accordance with institutional guidelines for the care and use of experimental animals and internationally accepted principles for the ethical use of animals in research.

All procedures involving the production of recombinant PpNPC2 and its experimental application were approved by the Committee on Biosafety, Faculty of Science, Mahidol University, Bangkok, Thailand (biosafety approval number MUSC 2023-A049). All recombinant DNA manipulation, bacterial transformation, protein expression, purification, and downstream experimental procedures were performed in compliance with institutional biosafety regulations.

Adult male *P. pelagicus* were obtained from a commercial aquaculture farm and acclimatized in aerated natural seawater before experimentation. The animals were humanely euthanized by immersion in 7.5% MgCl₂, followed by ice-water treatment before tissue collection to minimize tissue degradation. Every effort was made to minimize animal suffering and to use the minimum number of animals required to achieve scientifically valid results.

### Study period and location

Adult male blue swimming crabs were obtained from a commercial aquaculture farm in Samut Sakhon Province, Thailand. The experimental procedures were conducted at the Faculty of Science, Mahidol University, Bangkok, Thailand, from August 2022 to December 2025.

### Study design

This experimental study used sperm isolated from the testes and spermatic ducts of adult male blue swimming crabs. Sperm samples were assigned randomly to recombinant PpNPC2 treatment or control groups. Each experiment included sperm obtained from at least three independent animals as biological replicates, and all measurements were performed in duplicate or triplicate, as specified for each assay. Investigators conducting the analyses were blinded to the treatment allocation to minimize experimental bias.

### *PpNPC2* sequence comparison and molecular genetic analysis

Transcriptomic and proteomic data for *Portunus pelagicus NPC2* (*PpNPC2*) were obtained from the National Center for Biotechnology Information (NCBI, Bethesda, MD, USA) database under GenBank accession number PZ191617.1. The sequence was analyzed using the NCBI tBLASTn program (BLAST version 2.14.1+; https://blast.ncbi.nlm.nih.gov/Blast.cgi) to identify NPC2 homologs. Sequences with an identity >30% and query coverage >70% were selected for further analysis. The retrieved nucleotide sequence was translated into its corresponding amino acid sequence using the ExPASy translation tool (Swiss Institute of Bioinformatics, Lausanne, Switzerland; https://web.expasy.org/translate). The deduced protein sequence (protein accession number YFH55873.1) was characterized based on sequence similarity and homology with previously identified NPC2 proteins using Clustal Omega alignment software (version 1.2.4; European Bioinformatics Institute, Cambridge, UK; http://www.ebi.ac.uk/Tools/msa/clustalo/).

NPC2 amino acid sequences from representative species were retrieved from the NCBI database, including *Homo sapiens* (NP_006423.1), *Bos taurus* (NP_776343.1), *Canis lupus familiaris* (NP_001003242.2), *Mus musculus* (AAH07190.1), *Penaeus japonicus* (AKO69814.1), *Xenopus laevis* (AAH60392.1), *Danio rerio* (AAF99719.1), *Monodelphis domestica* (XP_005187474.1), *Aedes aegypti* (XP_001647805.2), *Daphnia pulex* (EFX84782.1), *Penaeus monodon* (ALG65704.1), *Litopenaeus* *vannamei* (XP_027225341.1), *Macrobrachium rosenbergii* (SRP049917), and *P. pelagicus* (YFH55873.1). Multiple sequence alignment was performed using the progressive alignment algorithm.

Phylogenetic analysis was performed using Molecular Evolutionary Genetics Analysis software (MEGA X, version 10.2.6; Institute for Genomics and Evolutionary Medicine, Temple University, Philadelphia, PA, USA). Phylogenetic relationships were inferred using the maximum likelihood method based on the Jones–Taylor–Thornton amino acid substitution model, with gamma-distributed rate variation among sites. The statistical reliability of the tree topology was evaluated using 1000 bootstrap replicates, and bootstrap values ≥70% were considered indicative of strong clade support.

The three-dimensional structure of PpNPC2 was predicted using UCSF ChimeraX software (version 1.9; University of California, San Francisco, CA, USA; http://www.cgl.ucsf.edu). Homology modeling was performed using the crystal structure of human NPC2 (Protein Data Bank accession 2HKA) as the template. Structural visualization and ligand-binding pocket analysis were subsequently performed within the ChimeraX environment.

### Recombinant PpNPC2 production and purification

Recombinant PpNPC2 was produced using a heterologous expression system in *Escherichia coli* with methodological modifications. This study represents the first reported heterologous bacterial expression and functional application of a brachyuran NPC2 protein to crab sperm.

The *PpNPC2* coding sequence was codon-optimized for bacterial expression (Supplementary Figure 1) and commercially synthesized by General Biosystems (Durham, NC, USA) based on transcriptomic data deposited under GenBank accession number PZ191617.1. The synthesized sequence was subcloned into the pET-17b expression vector (Novagen/Merck, Darmstadt, Germany). To facilitate downstream purification, the recombinant construct was designed to encode a C-terminal hexahistidine affinity tag.

The recombinant plasmid was transformed into competent *E. coli* BL21(DE3) cells (New England Biolabs, Ipswich, MA, USA). Transformants were confirmed using colony polymerase chain reaction and sequencing with the following primer pair: forward, 5′-TAATACGACTCACTATAGGG-3′; and reverse, 5′-ATGGCCAAGGTGCTCTTC-3′. Bacterial cultures were grown in Luria–Bertani broth supplemented with 50 µg/mL kanamycin at 37°C with shaking at 200 rpm until the optical density at 600 nm reached 0.6–0.8. Protein expression was induced by adding 0.6 mM isopropyl β-D-1-thiogalactopyranoside (Sigma-Aldrich, St. Louis, MO, USA), followed by incubation at 30°C for 6 h.

The bacterial pellet was collected by centrifugation at 6000 × *g* for 10 min at 4°C and resuspended in lysis buffer containing 100 mM Na₂HPO₄, 10 mM Tris-HCl (pH 8.0), 6 M guanidine hydrochloride, 8 M urea, 20 mM imidazole, and 1% Triton X-100. The cells were disrupted using a Vibra-Cell VCX130 probe sonicator (Sonics & Materials Inc., Newtown, CT, USA). Recombinant PpNPC2 was purified using nickel–nitrilotriacetic acid affinity chromatography with a HisTrap HP 1-mL column (GE Healthcare, Chicago, IL, USA) containing an approximate resin-bed volume of 1 mL. The column was equilibrated with binding buffer containing 50 mM Tris-HCl, 300 mM NaCl, and 20 mM imidazole at pH 8.0. The bound protein was eluted using a 20–250 mM imidazole gradient prepared in the same buffer. After denaturant removal, protein refolding was achieved by dialysis against phosphate-buffered saline (PBS; pH 7.4) at 4°C for 24 h, with three buffer changes.

The biochemical properties of PpNPC2 were evaluated to determine its concentration, purity, structural refolding, and endotoxin content. Protein concentration was determined using a Pierce Bicinchoninic acid (BCA) Protein Assay Kit (Thermo Fisher Scientific, Waltham, MA, USA) according to the manufacturer’s instructions. Protein purity was initially evaluated using 12.5% sodium dodecyl sulfate–polyacrylamide gel electrophoresis. Endotoxin concentrations were measured using a Limulus amebocyte lysate assay kit (Lonza, Basel, Switzerland) to confirm the suitability of the purified protein for subsequent biological assays.

Circular dichroism spectroscopy was performed using a JASCO J-815 spectropolarimeter (JASCO, Tokyo, Japan) to verify protein folding. Briefly, PpNPC2 at a concentration of 1.1 mg/mL was placed in a quartz cuvette with a path length of 0.1 cm. Spectra were recorded over the wavelength range 190–260 nm with a bandwidth of 1 nm and a scanning speed of 50 nm/min. Four technical replicates were analyzed. Spectral intensity was converted from instrumental millidegrees to Δε (M⁻¹ cm⁻¹) and analyzed using the BeStSel server [23]. To maintain protein stability, purified recombinant PpNPC2 was stored at −80°C in PBS containing 10% glycerol.

### Animals and sperm treatment

A total of 18 adult male *P. pelagicus* were used. The crabs had carapace widths of 12–15 cm and body weights of 250–350 g. Before experimentation, the crabs were maintained in aerated natural seawater for 24 h. Euthanasia was performed by immersion in 7.5% MgCl₂, followed by placement in ice-water to reduce the rate of tissue degradation.

The reproductive tract, including the testis and spermatic duct, was carefully dissected and cut into small fragments in Tris-buffered saline (TBS; pH 7.4). The tissues were gently homogenized using a glass-rod homogenizer and filtered through a 45-µm mesh filter (Endecotts Ltd., London, UK) to remove tissue debris. The filtrate was centrifuged at 1500 × *g* for 5 min at 4°C to obtain sperm pellets.

The sperm were resuspended in artificial seawater (ASW) containing 450 mM NaCl, 10 mM KCl, 10 mM CaCl₂, 30 mM MgCl₂, and 10 mM 4-(2-Hydroxyethyl)-1-piperazineethanesulfonic acid (HEPES) buffer. The ASW was adjusted to pH 7.8 and an osmolarity of 950–980 mOsm/kg. The final sperm concentration was adjusted to 1 × 10⁶ cells/mL, as determined manually using a hemocytometer. Sperm viability was evaluated before and after treatment using 0.4% trypan blue exclusion staining and light microscopy. Samples with >90% viable sperm were included in subsequent experiments.

Sperm isolated from the testis and spermatic duct were treated with recombinant PpNPC2 at concentrations of 50–100 µg/mL. Sperm incubated in ASW alone served as the control. Samples were incubated at room temperature (25°C) for 30 min with gentle agitation at 100 rpm using an orbital shaker to ensure uniform exposure to the recombinant protein. After incubation, the sperm samples were washed twice with ASW by centrifugation at 1500 × *g* for 5 min at 4°C. The recovered sperm were subsequently used for lipid analysis, protein expression analysis, immunofluorescence microscopy, and evaluation of sperm morphological remodeling.

Each experiment was performed using sperm obtained from at least three independent animals as biological replicates. All measurements were performed in technical duplicate. Samples were randomly assigned to the treatment groups, and investigators performing the analyses were blinded to the treatment conditions to minimize experimental bias.

### Neutral lipid and fatty acid analyses

Lipids were extracted from 2 × 10⁷ testicular sperm (Tsp) and spermatic duct sperm (Ssp) suspended in 1 mL of ASW using a chloroform–methanol–water extraction procedure [24]. Neutral lipid fractions were subsequently analyzed using high-performance thin-layer chromatography (HPTLC) on F₂₅₆ silica gel plates (Merck, Darmstadt, Germany). Chromatographic separation was performed using a solvent mixture of n-hexane, diethyl ether, and acetic acid at a volumetric ratio of 70:30:2.

The lipid bands were visualized by staining for 10 min with 0.1% weight/volume Coomassie Brilliant Blue G prepared in methanol, water, and acetic acid at a volumetric ratio of 50:40:10. The plates were destained with methanol until a clear background was obtained. CHO standards ranging from 1 to 10 µg were used to generate a calibration curve. Densitometric analysis of the CHO bands was performed using ImageJ software (version 1.8.0; National Institutes of Health, Bethesda, MD, USA). Band intensities were quantified after background subtraction, and the CHO content was calculated using the standard calibration curve.

For fatty acid analysis, fatty acid methyl esters (FAMEs) were prepared by acid-catalyzed transesterification using methanolic HCl. The FAMEs were analyzed using gas chromatography–mass spectrometry (GC–MS) with an Agilent 6980 gas chromatograph coupled to an Agilent 5973 Mass Selective Detector (Agilent Technologies, Santa Clara, CA, USA). Separation was performed using a DB-Innowax capillary column (30 m × 0.25 mm, 0.25 µm film thickness; Agilent Technologies). Helium was used as the carrier gas in split mode at a 1:10 ratio.

The injector temperature was maintained at 250°C, and the ion-source temperature was maintained at 230°C. Mass spectra were recorded in electron-ionization mode over a mass-to-charge ratio range of 50–550. The oven temperature was initially maintained at 80°C for 1 min, then increased to 175°C at 5°C/min, to 250°C at 4°C/min, and finally held for 10 min.

Peaks were identified by comparison with a certified Supelco 37 Component FAME Mix (Sigma-Aldrich). Fatty acids were quantified using MassHunter Quantitative Analysis software (version B.07.00; Agilent Technologies) based on peak integration relative to the internal standard, nonadecanoic acid (C19:0).

All lipid measurements were obtained from three independent biological replicates (n = 3), with each sample analyzed in technical duplicate. Statistical comparisons were performed using one-way analysis of variance (ANOVA), followed by Tukey’s post hoc test. The data were expressed as the mean ± standard error of the mean.

### Localization of CHO using Filipin III staining

To determine the localization of CHO in sperm membranes, live Tsp and Ssp were stained with Filipin III, a fluorescent CHO-binding polyene antibiotic derived from *Streptomyces filipinensis* [25]. A Filipin III stock solution at a concentration of 1 mg/mL was prepared in dimethyl sulfoxide, divided into aliquots, protected from light, and stored at −20°C. Fresh working solutions were prepared by diluting the stock solution in TBS to a final concentration of 1 µg/mL.

The sperm suspensions were incubated with Filipin III for 1 h at 25°C in the dark. After staining, the sperm were washed twice with TBS by centrifugation at 1500 × *g* for 5 min at 4°C to remove unbound dye. The sperm pellets were resuspended in a 1:1 volumetric mixture of TBS and glycerol for mounting.

Fluorescence images were acquired using a MICA fluorescence microscope (Leica Microsystems, Wetzlar, Germany). Filipin fluorescence was visualized using an excitation wavelength of 340–380 nm and an emission wavelength of 385–470 nm. Exposure time and gain settings were held constant across all samples to enable quantitative comparisons.

Fluorescence intensity was quantified using ImageJ software. Regions surrounding individual sperm heads were manually selected, with at least 50 sperm analyzed per sample. The mean fluorescence intensity was measured after background subtraction using the rolling-ball algorithm.

All experiments were performed using sperm obtained from three independent animals as biological replicates, with each measurement conducted in technical triplicate. Differences in fluorescence intensity among groups were analyzed using one-way ANOVA, followed by Tukey’s post hoc test. Statistical significance was set at p < 0.05.

### Immunohistochemistry in the tissues and immunofluorescence of the isolated sperm

Testis and spermatic duct tissues were fixed in 4% paraformaldehyde at 4°C overnight, embedded in paraffin, and sectioned at a thickness of 5–7 µm using a rotary microtome (Leica RM2235, Leica Microsystems, Wetzlar, Germany). Tissue sections were deparaffinized in xylene, rehydrated through a graded ethanol series, and rinsed with TBS. Antigen retrieval was performed by heating the sections in 10 mM citrate buffer (pH 6.0) at 95°C for 15 min in a water bath, followed by cooling to 25°C. The sections were permeabilized with 1% Triton X-100 in TBS for 15 min and blocked with 4% bovine serum albumin (BSA) in TBS for 3 h at 25°C. The sections were then incubated overnight at 4°C with anti-MrNPC2 polyclonal antibody (1 µg/mL; Sa-bio, Singapore) diluted in blocking buffer (final incubation volume, 100 µL per section). After three washes with TBS (5 min each), the sections were incubated with Horseradish peroxidase (HRP)-conjugated goat anti-rabbit immunoglobulin (Ig)G secondary antibody (1:200 dilution; Abcam) for 2 h at 25°C. Immunoreactivity was visualized using a nickel-enhanced 3,3′-Diaminobenzidine (DAB) substrate (Vector Laboratories, Burlingame, CA, USA). Color development was monitored microscopically and terminated after 3–5 min by rinsing the sections with distilled water to prevent over-staining. The sections were counterstained with hematoxylin, dehydrated, mounted, and examined using an Olympus BX53 light microscope (Olympus Corporation, Tokyo, Japan). Antibody titration (0.1–10 µg/mL), cross-reactivity with recombinant PpNPC2 protein, and antibody pre-absorption with PpNPC2 protein were validated by Western blotting before immunohistochemical application. Rabbit IgG isotype control (10 µg/mL; Abcam) was used as a negative control.

For sperm immunofluorescence analysis, Tsp and Ssp were fixed with 4% paraformaldehyde for 20 min at 25°C and washed with TBS. The samples were blocked with 5% BSA in TBS for 1 h and incubated overnight at 4°C with monoclonal anti-phosphotyrosine antibody (1:200 dilution; Abcam; final incubation volume, 200 µL per sample). Following incubation, the samples were washed three times with TBS containing 0.05% Tween-20 (5 min per wash). The sperm were then incubated with goat anti-mouse IgG conjugated with Alexa Fluor 488 (1:400 dilution; Molecular Probes, Eugene, OR, USA) for 1 h at 25°C in the dark. The nuclei were counterstained with 1 µg/mL 4′,6-Diamidino-2-phenylindole (DAPI) for 5 min. Fluorescence images were acquired using a Leica DM6 B fluorescence microscope (Leica Microsystems, Wetzlar, Germany). Image acquisition parameters, including laser power, detector gain, and exposure time, were maintained constant for all samples to enable quantitative comparison. All immunostaining experiments were performed using tissues or sperm obtained from three independent animals (biological replicates), and each staining procedure was repeated in technical triplicate.

### Western blotting

Proteins from Tsp, Ssp, and PpNPC2-treated groups were extracted in TBS containing protease and phosphatase inhibitor cocktails (Roche Diagnostics, Mannheim, Germany). Protein extraction was performed on ice for 30 min with intermittent vortexing, followed by centrifugation at 12,000 × *g* for 15 min at 4°C to obtain clarified lysates. Protein concentrations were determined using a Pierce™ BCA Protein Assay Kit (Thermo Fisher Scientific, Waltham, USA). Equal amounts of protein (20 µg per lane) were mixed with Laemmli sample buffer containing 5% β-mercaptoethanol, heated at 95°C for 5 min, and separated on 10% SDS-polyacrylamide gels under reducing conditions.

Proteins were transferred onto polyvinylidene difluoride membranes (0.45-µm pore size; Millipore, Bedford, MA, USA) using a wet transfer system (Bio-Rad Mini Trans-Blot, Hercules, CA, USA) at 100 V for 90 min at 4°C in transfer buffer containing 25 mM Tris, 192 mM glycine, and 20% methanol. The membranes were blocked with 4% BSA and 2% skim milk in TBS (pH 7.6) for 2 h at 25°C, then incubated overnight at 4°C with primary antibodies diluted in blocking buffer. The primary antibodies included monoclonal anti-phosphotyrosine antibody (Abcam; catalog no. ab179530; 1:1000 dilution) and anti-β-actin antibody (Cell Signaling Technology, Danvers, MA, USA; catalog no. 4970; 1:2000 dilution), which served as the loading control. After washing three times with Tris-buffered saline with Tween-20 for 10 min each, the membranes were incubated with HRP-conjugated goat anti-mouse or goat anti-rabbit secondary antibodies (Abcam; catalog nos. ab205719 and ab205718; 1:5000 dilution) for 1 h at 25°C. Protein bands were visualized using an ECL detection kit (Clarity™ Western ECL Substrate, Bio-Rad, Hercules, CA, USA). Chemiluminescent signals were captured using a ChemiDoc MP Imaging System (Bio-Rad) with exposure times ranging from 10 to 60 s to avoid signal saturation. Band intensities were quantified by densitometric analysis using ImageJ software (version 1.8.0), and phosphotyrosine-positive signals were normalized to the β-actin band. All experiments were performed using protein extracts from three independent animals (biological replicates), and each Western blot was performed in technical duplicate.

### Induction of sperm capacitation by PpNPC2 and sperm morphological analysis

The induction of PpNPC2-mediated sperm capacitation-like changes was adapted from the previously described prawn system [16] with minor modifications. Approximately 1 × 10⁵ Tsp and Ssp were resuspended in 200 µL TBS and treated with recombinant PpNPC2 (50–100 µg/mL) for 10, 30, or 60 min at 25°C with gentle agitation (100 rpm). Following incubation, the sperm were centrifuged at 1,600 × *g* for 5 min and fixed with 2% paraformaldehyde in TBS for 30 min at 25°C. The nuclei were counterstained with 1 µg/mL DAPI. Images were captured using a Leica DM6 B fluorescence microscope equipped with differential interference contrast optics under the conditions described above.

The morphological characteristics of Tsp and Ssp were classified according to the previously reported brachyuran crab sperm morphology [22] using a simplified lettering system (Type 1-3). Type 1 sperm exhibited a typical round morphology characterized by a centrally located dense acrosomal vesicle surrounded by DAPI-stained nuclear material. Type 2 sperm exhibited eccentric displacement of the acrosomal vesicle with partial protrusion of the acrosomal granule or filament. Type 3 sperm exhibited a distinct snowman-like morphology in which the acrosomal vesicle was completely protruded and detached from the nuclear region. Approximately 200 sperm cells per sample were evaluated from at least eight random microscopic fields. Morphological scoring was independently performed by two blinded observers, and inter-observer agreement was assessed using Cohen’s kappa coefficient (κ = 0.86). Sample size was determined using *G*Power 3.1 (Informer Technologies Inc., Raleigh, NC, USA) with the following analysis parameters: power = 0.80, α = 0.05, and effect size = 0.35. All experiments were performed using sperm obtained from three independent animals (biological replicates), and each treatment condition was analyzed in technical triplicate. Data were expressed as mean ± SD. Statistical analysis was performed using IBM SPSS Statistics software (version 26.0; IBM Corp., Armonk, NY, USA). Data normality was assessed using the Shapiro–Wilk test before applying one-way ANOVA, followed by Tukey’s post hoc test for multiple comparisons.

## RESULTS

### Characterization of PpNPC2

The male reproductive system consists of paired testes, responsible for sperm production, and spermatic ducts, involved in sperm transport and release during mating. To examine whether a capacitation-like process occurs in brachyuran sperm, three physiological parameters were evaluated: CHO efflux, inducible Protein tyrosine phosphorylation (PTP), and sperm morphological alterations.

**Figure 1:**
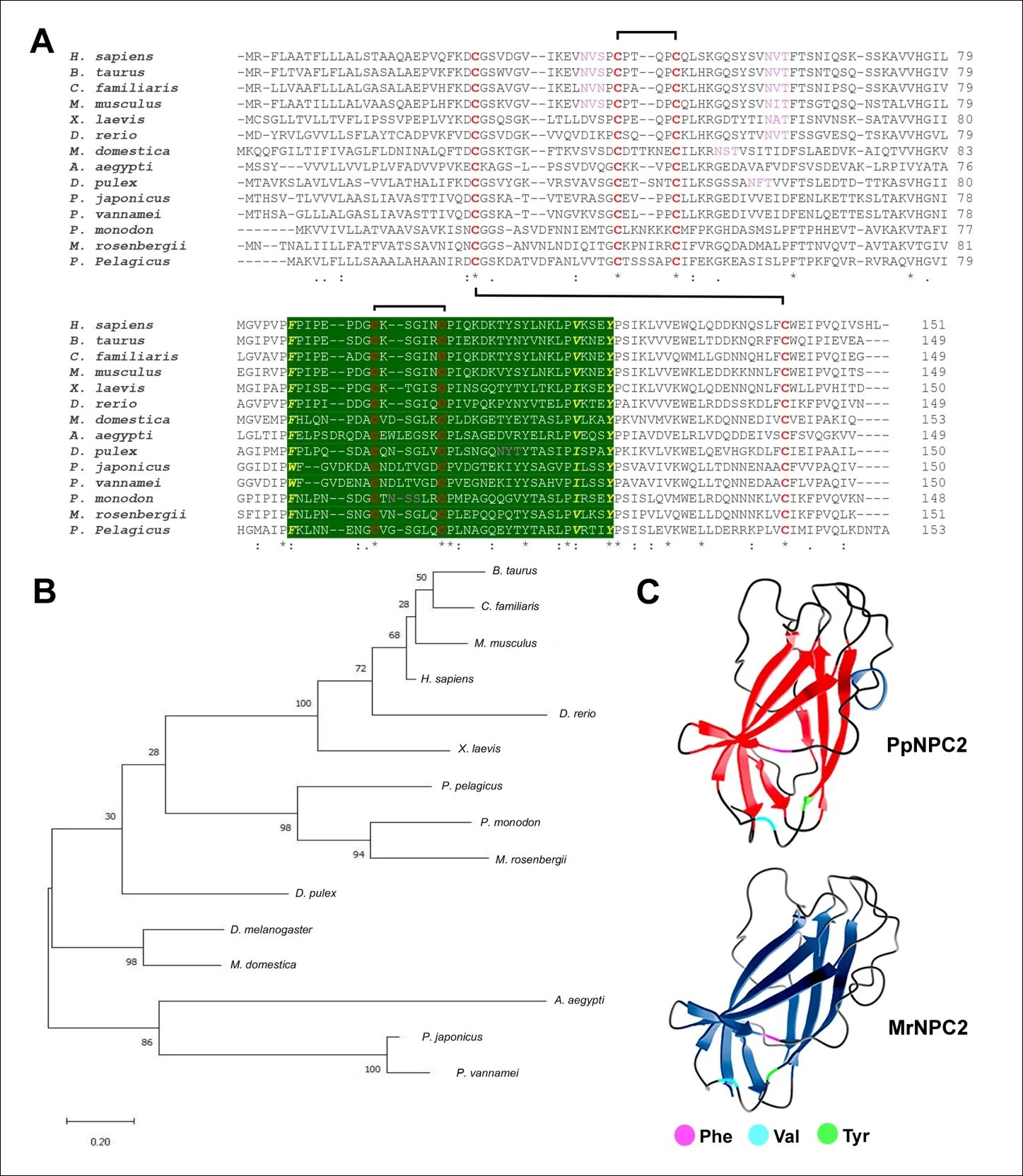
Sequence characterization, phylogenetic analysis, and structural prediction of PpNPC2. (A) Multiple sequence alignment of PpNPC2 with representative NPC2 homologs showing the six conserved cysteine residues (red, bold), conserved CHO-binding gate residues (F, I/V, and Y; yellow letters within the green box), and predicted N-glycosylation motif (pink). (B) Phylogenetic tree illustrating the evolutionary relationship between PpNPC2 and NPC2 proteins from representative vertebrate and invertebrate species. (C) Predicted three-dimensional ribbon structure of PpNPC2 highlighting the CHO-binding opening gate. PpNPC2 represents the first characterized brachyuran NPC2 and clusters within the crustacean clade, showing a close evolutionary relationship with shrimp NPC2 homologs. CHO = cholesterol; NPC2 = Niemann–Pick type C2; PpNPC2 = *Portunus pelagicus* Niemann–Pick type C2.

The full-length PpNPC2 sequence comprised 651 nucleotides and was deposited in GenBank (accession no. PZ191617.1). The corresponding open reading frame encoded a protein of 153 amino acid residues (protein accession no. YFH55873.1). The predicted molecular mass of PpNPC2 was 16.64 kDa, with a predicted pI of 8.99. Six highly conserved cysteine residues (Cys23, Cys40, Cys79, Cys94, Cys101, and Cys142), predicted to form three intramolecular disulfide bonds, were identified, representing a characteristic feature of NPC2 proteins (Figure 1A, solid lines). In addition, a conserved CHO-binding region (green box) and three amino acid residues (Phe86, Val117, and Tyr121) forming the structural opening of the CHO-binding pocket were identified. A conserved N-linked glycosylation site (pink letters) was also identified in the PpNPC2 sequence (Figure 1A). Comparative sequence analysis with NPC2 proteins from 14 representative species demonstrated that PpNPC2 shared the highest sequence identity with crustacean NPC2 homologs, including *Macrobrachium rosenbergii* (60.3%), *Penaeus monodon* (64.1%), and *Litopenaeus* *vannamei* (64.3%). Lower sequence identities were observed with vertebrate NPC2 proteins, including *Homo sapiens* (55.4%) and *Bos taurus* (54.8%) (Figure 1A).

Phylogenetic analysis demonstrated that PpNPC2 clustered within the crustacean NPC2 clade and exhibited close evolutionary relationships with shrimp NPC2 homologs (Figure 1B). The distinct branch lengths separating PpNPC2 from shrimp NPC2 proteins suggest brachyuran-specific adaptations in sterol-binding affinity. Furthermore, 3D structural modeling demonstrated that PpNPC2 possesses the characteristic β-sheet-rich fold of the NPC2 protein family and contains NPC-like, ML (MD-2-related lipid-recognition), and Der-p2-like domains. Structural visualization further showed that the conserved residues were positioned at the entrance of a hydrophobic pocket, consistent with a putative CHO-binding site (Figure 1C).

### Localization of PpNPC2 in the male reproductive organs

**Figure 2:**
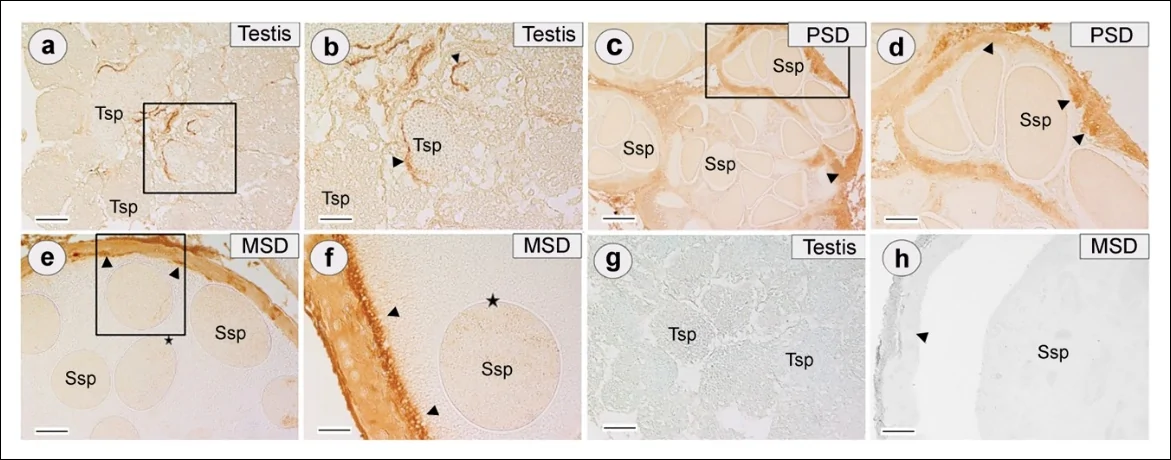
Immunolocalization of PpNPC2 in the testis and spermatic duct. Aldehyde-fixed sections of the testis and spermatic duct were immunostained with polyclonal anti-NPC2 antibody followed by an HRP-conjugated secondary antibody and examined under a light microscope. Positive immunoreactivity was detected predominantly in the surrounding epithelial cells of the testis (a and b, arrowheads), PSD (c and d, arrowheads), and MSD (e and f, arrowheads). In contrast, Tsp and Ssp exhibited minimal immunoreactivity, whereas the negative controls showed no detectable staining (g and h). Scale bars = 50 µm. HRP = horseradish peroxidase; MSD = middle spermatic duct; NPC2 = Niemann–Pick type C2; PpNPC2 = *Portunus pelagicus* Niemann–Pick type C2; PSD = proximal spermatic duct; Ssp = spermatic duct sperm; Tsp = testicular sperm.

**Figure 3:**
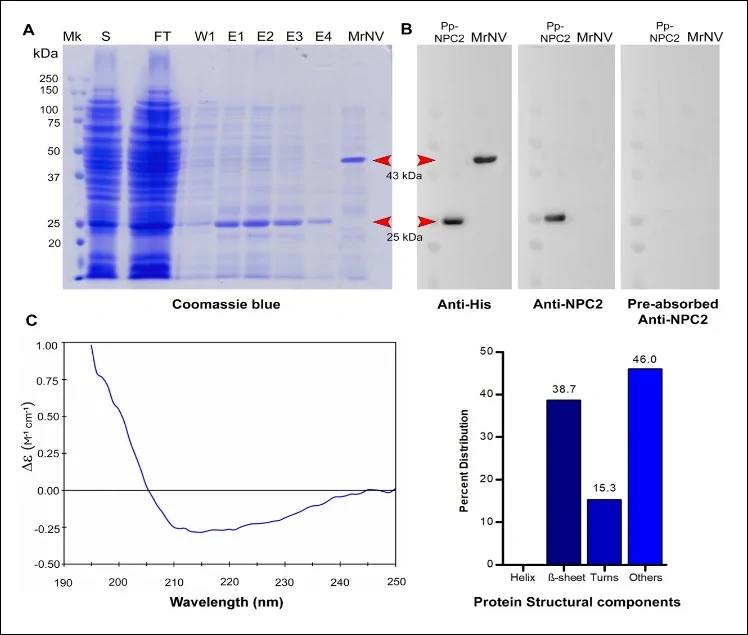
Expression, purification, and characterization of recombinant PpNPC2. Recombinant PpNPC2 was expressed in *Escherichia coli* BL21 carrying the pET-17b-PpNPC2 expression construct and purified by nickel–nitrilotriacetic acid affinity chromatography. (A) SDS-PAGE analysis of the purification procedure showing the supernatant (S), flow-through (FL), wash (W), and elution fractions (E1–E4). (B) Western blot analysis using anti-hexahistidine antibody (left), anti-NPC2 antibody (middle), and anti-NPC2 antibody pre-absorbed with a 10-fold excess of recombinant PpNPC2 (right). Arrowheads indicate recombinant PpNPC2 (25 kDa) and MrNV capsid protein (43 kDa). (**C**) Circular dichroism spectrum analyzed using the BeStSel server together with the predicted secondary structural components of recombinant PpNPC2. E = elution fraction; FL = flow-through fraction; MrNV = *Macrobrachium rosenbergii* nodavirus; NPC2 = Niemann–Pick type C2; PpNPC2 = *Portunus pelagicus* Niemann–Pick type C2; S = supernatant; SDS-PAGE = sodium dodecyl sulfate-polyacrylamide gel electrophoresis; W = wash fraction.

Localization of PpNPC2 in the male reproductive tract was examined by immunohistochemistry using the affinity-purified polyclonal anti-NPC2 antibody. As shown in Figure 2, PpNPC2 immunoreactivity was detected in the testis and in both the proximal and middle regions of the spermatic duct. In the testis, positive immunostaining was predominantly observed in supporting epithelial (nurse) cells (Figure 2a), whereas germ cells within the luminal compartments showed no immunoreactivity (Figure 2b). Similarly, strong cytoplasmic immunoreactivity was detected in the epithelial cells lining the spermatic duct (Figures 2c–f). Negative control sections incubated with rabbit IgG isotype control or without the primary antibody showed no detectable immunoreactivity in either the testis or spermatic duct (Figures 2g and h), confirming the specificity of the staining. These findings demonstrate for the first time that NPC2 is expressed in the epithelial cells, but not in sperm, within the male reproductive tract. It should be noted that the anti-NPC2 antibody used in this study was verified for specificity and optimal concentration using purified recombinant PpNPC2, as shown in Figure 3.

### Production of recombinant PpNPC2 protein

Recombinant PpNPC2 was expressed in *Escherichia coli* and purified by nickel affinity chromatography for subsequent functional assays. As shown in Figure 3A, Coomassie Brilliant Blue staining revealed distinct protein profiles throughout the purification procedure, including the supernatant (lane S), flow-through fraction (lane FT), wash fraction (lane W), and eluted fractions (lanes E2–E4). A prominent protein band of approximately 25 kDa was observed in the eluted fractions, indicating successful enrichment of recombinant PpNPC2. Densitometric analysis of the stained gels estimated the purity of the recombinant protein to be >95%. Western blot analysis using an anti-His antibody detected a single immunoreactive band at approximately 25 kDa in all protein fractions analyzed (Figure 3B), confirming successful expression and purification of the His-tagged recombinant PpNPC2. The Limulus amebocyte lysate assay demonstrated a low endotoxin level (0.2–0.3 EU/mL), indicating that the purified recombinant protein was suitable for subsequent sperm functional assays.

Purified PpNPC2 was subsequently used to evaluate the specificity of the affinity-purified polyclonal anti-NPC2 antibody and to determine the optimal antibody concentration for Western blotting, immunohisto-chemistry, and CHO-binding assays. The antibody showed strong reactivity toward purified PpNPC2, with the optimal concentration determined to be 10 µg/mL, producing the strongest immunoreactive band at approximately 25 kDa among the concentrations tested (Supplementary Figure 2). In contrast, the irrelevant control protein (MrNV capsid protein carrying a hexahistidine tag) and the pre-absorbed anti-NPC2 antibody (pre-incubated with 100 µg/mL PpNPC2) showed negligible immunoreactivity (Figure 3B and C). Dot blot analysis demonstrated specific binding of PpNPC2 to CHO but not to the other lipid species examined (Supplementary Figure 3, upper panel). Lipid extracts prepared from both Tsp and Ssp exhibited strong and moderate PpNPC2 immunoreactivity, respectively, which was effectively inhibited by the anti-NPC2 antibody (Supplementary Figure 3, middle panel). In addition, circular dichroism spectroscopy was performed to verify the structural folding of PpNPC2. Spectral analysis revealed that the protein comprised 38.7% β-sheet, 15.3% turns, and 46.0% unordered structures, with no detectable α-helical content (Figures 3D and E). The low NRMSD (<0.1) indicated a highly reliable structural prediction.

### Neutral lipid and fatty acid profiles of Tsp and Ssp

**Figure 4:**
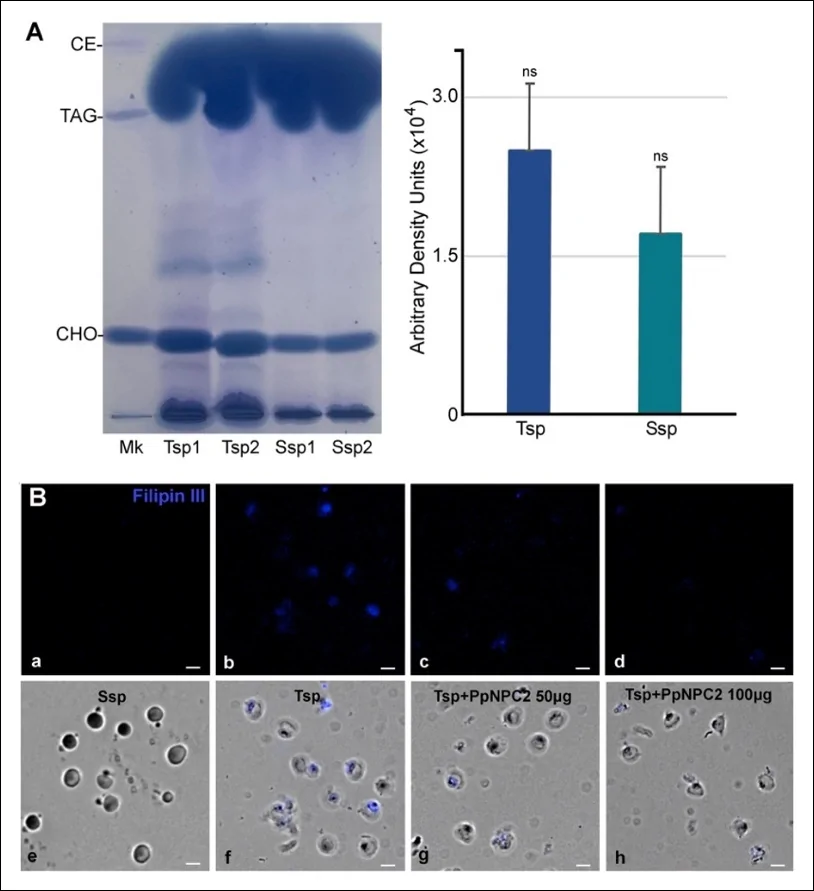
Neutral lipid analysis and membrane CHO localization in crab sperm. (A) Neutral lipids extracted from Tsp and Ssp were separated by HPTLC using an *n*-hexane:diethyl ether:acetic acid (70:30:2, v/v/v) solvent system and visualized by Coomassie Brilliant Blue staining. CHO band intensities were quantified by densitometric analysis using ImageJ software (right). (B) Membrane CHO localization determined by Filipin III staining. Live Tsp and Ssp, with or without PpNPC2 treatment, were stained with Filipin III and examined under a fluorescence microscope (upper panels) and DIC microscope (lower panels). Scale bar = 10 µm. CHO = cholesterol; DIC = differential interference contrast; HPTLC = high-performance thin-layer chromatography; PpNPC2 = *Portunus pelagicus* Niemann–Pick type C2; Ssp = spermatic duct sperm; Tsp = testicular sperm.

The neutral lipid and fatty acid compositions of Tsp and Ssp were analyzed by HPTLC and gas chromatography–mass spectrometry (GC–MS), whereas sperm membrane CHO distribution was evaluated by Filipin III staining. HPTLC using the *n*-hexane/diethyl ether/acetic acid solvent system successfully resolved the major neutral lipid components in both sperm populations, including cerebroside (CE), triacylglycerol (TAG), and CHO (Figure 4A). Densitometric analysis demonstrated that CHO content was lower in Ssp than in Tsp, with mean intensity values of 1.7 × 10⁴ and 2.5 × 10⁴ arbitrary units, respectively. This corresponded to an approximately 32% reduction in CHO during sperm transit through the spermatic duct, although the difference was not statistically significant (p = 0.28) (Figure 4A). Consistent with these findings, Filipin III staining showed strong membrane-associated fluorescence in Tsp, whereas markedly weaker fluorescence was observed in Ssp, indicating reduced membrane CHO content. Similarly, treatment of Tsp with recombinant PpNPC2 resulted in a progressive decline in Filipin III fluorescence intensity, with the 100 µg/mL PpNPC2 treatment producing the greatest reduction, closely resembling the staining pattern observed in Ssp (Figure 4B).

GC–MS analysis (Table 1) demonstrated that unsaturated fatty acids were the predominant lipid class in Tsp, accounting for approximately 60% of the total fatty acids, whereas their proportion decreased in Ssp. Polyunsaturated fatty acids (PUFAs) were more abundant in Tsp (51.54 ± 2.35 arbitrary units) than in Ssp (40.82 ± 7.03 arbitrary units), representing a 21% reduction during sperm maturation (p = 0.08). DHA, the predominant PUFA, was detected at higher levels in Tsp (25.14 ± 3.08 arbitrary units) than in Ssp (16.78 ± 4.77 arbitrary units), corresponding to an approximately 1.5-fold decrease (p = 0.09). Monounsaturated fatty acids (MUFAs) remained relatively stable between the two sperm populations, with oleic acid (C18:1n9) as the predominant MUFA, decreasing by approximately 5% from Tsp (15.98 ± 1.25 arbitrary units) to Ssp (15.14 ± 2.84 arbitrary units) (p = 0.84). In contrast, saturated fatty acids increased during sperm transit, rising from 27.21 ± 2.77 arbitrary units in Tsp to 39.21 ± 2.09 arbitrary units in Ssp, representing a significant 31% increase (p < 0.005). Stearic acid (C18:0) remained the predominant saturated fatty acid, although its abundance did not differ significantly between Tsp (18.10 ± 2.36 arbitrary units) and Ssp (14.85 ± 1.53 arbitrary units) (p = 0.12).

**Table 1:** Fatty acid compositions of the freely isolated sperm from the testis and spermatic duct.

**Fatty acid species**	**Tsp (Mean ± SD)**	**Ssp** ** (Mean ± SD)**	**p** ***-*** **value**
C12:0 (Lauric acid)	0 ± 0	3.67 ± 0.77	0.0012
C14:0 (Myristic acid)	0 ± 0	6.69 ± 1.28	0.0008
C16:0 (Palmitic acid)	7.82 ± 0.51	13.90 ± 2.80	0.0223
C16:1n7 (Palmitoleic acid)	2.15 ± 2.15	0 ± 0	0.0005
C17:0 (Margaric acid)	1.97 ± 0.58	0 ± 0	0.0066
C18:0 (Stearic acid)	18.1 ± 2.36	14.85 ± 1.53	0.1164
C18:1n9 (Oleic acid)	15.98 ± 1.25	15.14 ± 2.84	0.8629
C18:1n7 (Vaccenic acid)	2.61 ± 0.21	4.91 ± 4.96	0.3072
C20:4n6 (Arachidonic acid)	13.19 ± 1.34	9.53 ± 1.77	0.0462
C20:5n3 (EPA)	11.56 ± 0.39	14.51 ± 2.73	0.1379
C22:6n3 (DHA)	25.14 ± 3.08	16.78 ± 4.77	0.085
Total SAT	27.21 ± 2.77	39.12 ± 2.09	0.0048
Total MUFA	21.25 ± 0.99	20.05 ± 6.90	0.8416
Total PUFA	51.54 ± 2.35	40.82 ± 7.03	0.0777

Tsp = Testicular sperm; Ssp = Spermatic duct sperm; SD = Standard deviation; SAT = Saturated fatty acids; MUFA = Monounsaturated fatty acids; PUFA = Polyunsaturated fatty acids; EPA = Eicosapentaenoic acid; DHA = Docosahexaenoic acid; p = Probability value.

### Induction of Tsp morphological changes by PpNPC2

**Figure 5:**
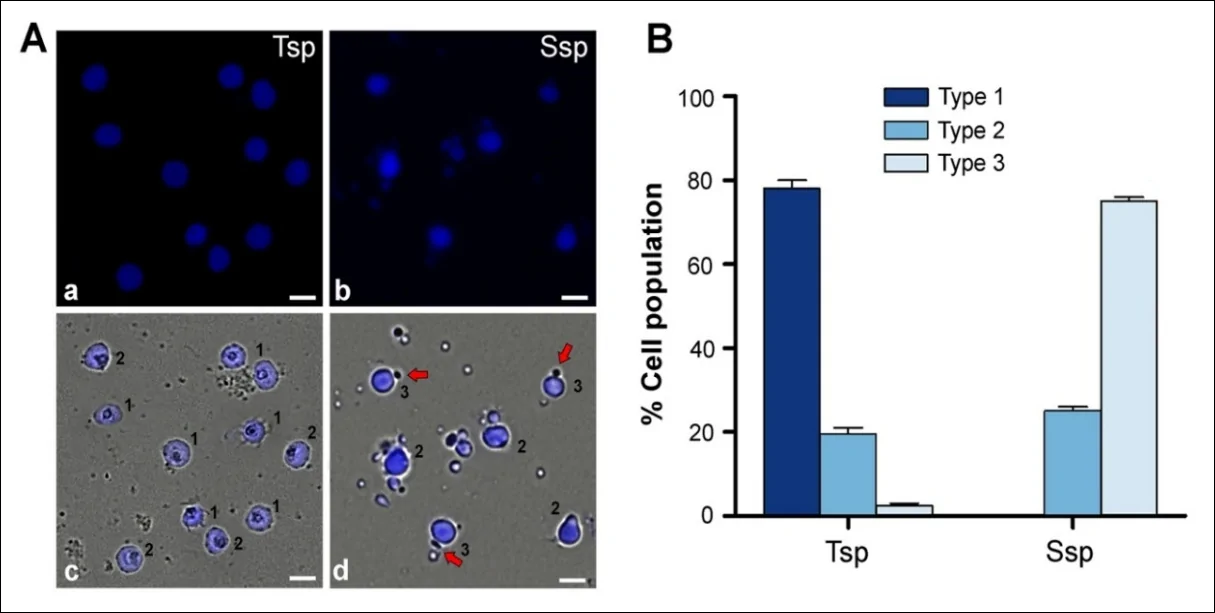
Morphological comparison of Tsp and Ssp. Aldehyde-fixed Tsp and Ssp were stained with DAPI (A) and classified into types 1, 2, and 3 according to the morphological criteria described in the Results section. Arrows indicate the characteristic snowman-like morphology unique to brachyuran sperm. (B) Distribution of the three sperm types in Tsp and Ssp. Scale bar = 10 µm. DAPI = 4′,6-diamidino-2-phenylindole; Ssp = spermatic duct sperm; Tsp = testicular sperm.

The morphological characteristics of Tsp and Ssp were first compared according to the previously reported brachyuran crab sperm morphology [22], with minor modifications to simplify sperm classification. Subsequently, the ability of recombinant PpNPC2 to induce comparable morphological changes in Tsp was evaluated. Most untreated Tsp (80.5% ± 2.83) exhibited the typical round morphology (type 1 sperm) (Figure 5A), whereas the remaining cells (19.5% ± 2.12) were classified as type 2 sperm. In contrast, most Ssp exhibited the characteristic snowman-like morphology (type 3 sperm), accounting for 75.0% ± 1.41 of the sperm population (Figure 5A and B). The remaining Ssp (25.0% ± 1.41) consisted of type 2 sperm, whereas type 1 sperm were not detected, indicating the complete disappearance of type 1 sperm during transit through the spermatic duct.

**Figure 6:**
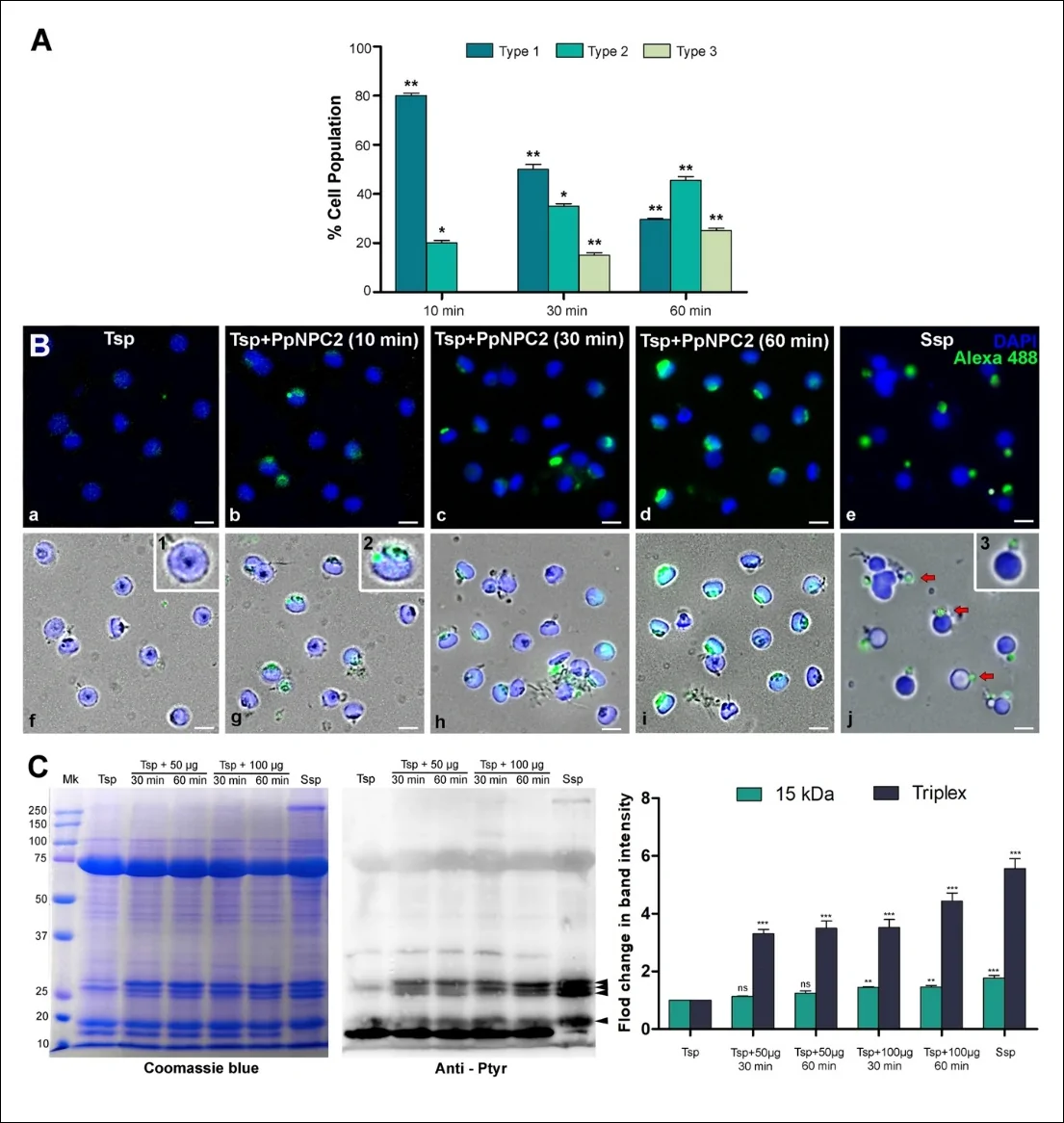
PpNPC2-induced morphological remodeling of Tsp and increased PTP. Tsp were incubated with 50–100 µg/mL recombinant PpNPC2 for 10–60 min. (A) Quantitative analysis of sperm morphology following PpNPC2 treatment. (B) Immunofluorescence analysis of PTP. Red arrows indicate the characteristic snowman-like morphology, and the insets show higher magnifications of each sperm type. (**C**) Western blot analysis demonstrating increased PTP in PpNPC2-treated Tsp and Ssp samples (arrowheads). The right panel shows densitometric analysis of the 15 kDa band and the 27/28/30 kDa protein triplet. Scale bar = 10 µm. PpNPC2 = *Portunus pelagicus* Niemann–Pick type C2; PTP = protein tyrosine phosphorylation; Ssp = spermatic duct sperm; Tsp = testicular sperm.

To determine whether PpNPC2 could induce comparable morphological remodeling, Tsp were incubated with recombinant PpNPC2 and examined over time. Treatment with 50 µg/mL PpNPC2 resulted in a progressive increase in type 2 sperm, from 19.5% in untreated samples to 22.5% ± 4.95 after 10 min (1.15-fold increase, p < 0.05), 34.0% ± 5.66 after 30 min (1.74-fold increase, p < 0.05), and 45.5% ± 2.12 after 60 min (2.33-fold increase, p < 0.01) (Figure 6A). Type 3 sperm first appeared after 30 min of incubation (16.0% ± 2.83, p < 0.01) and further increased to 26.5% ± 4.95 after 60 min (p < 0.01), demonstrating a time-dependent morphological transformation (Figure 6A). The conversion of Tsp from the typical round morphology to the snowman-like morphology following PpNPC2 treatment, or naturally during transit through the spermatic duct, represents a unique hallmark of brachyuran sperm capacitation and corresponds to the process previously described as acrosomal reversion in *Eriocheir sinensis* [17]. Regardless of its nomenclature, this process appears essential for sperm activation in the male reproductive tract, enabling sperm to acquire fertilizing competence.

Immunofluorescence analysis showed no detectable phosphotyrosine immunoreactivity in untreated Tsp (Figure 6B). Following PpNPC2 treatment, phosphotyrosine immunoreactivity increased markedly and was initially localized to the centrally positioned acrosomal granule of type 1 sperm. As morphological transformation progressed, the phosphotyrosine signal redistributed laterally and into the protruding acrosomal region of type 2 and type 3 sperm, closely resembling the staining pattern observed in Ssp (Figure 6B). Consistent with these observations, Western blot analysis demonstrated a progressive increase in phosphotyrosine-reactive protein bands, particularly those at approximately 15, 27, 28, and 30 kDa, in both concentration-dependent and time-dependent manners (Figure 6C). Collectively, recombinant PpNPC2 induced progressive structural remodeling of Tsp accompanied by increased PTP, indicating functional biochemical changes associated with sperm maturation.

## DISCUSSION

### NPC2 mediates a bicarbonate-independent capacitation-like pathway in brachyuran sperm

This study provides the first evidence that NPC2-mediated CHO sequestration operates in brachyuran crabs, revealing both conserved and divergent mechanisms of sperm activation across Decapoda. Physiologically, our findings demonstrate that NPC2 is associated with the complex, non-motile acrosomal remodeling characteristic of brachyuran sperm during the capacitation-like process. In mammals, sperm activation during capacitation is primarily regulated through two major mechanisms: bicarbonate influx and CHO sequestration [6, 26]. These mechanisms may function cooperatively or independently depending on the physicochemical environment surrounding the spermatozoa. In the bicarbonate-dependent pathway, bicarbonate enters sperm through sodium-bicarbonate cotransporters, activates soluble adenylyl cyclase, increases cyclic adenosine monophosphate production, stimulates protein kinase A, enhances membrane fluidity, and promotes progressive CHO efflux from the sperm membrane [8, 26, 27]. This signaling cascade subsequently induces lipid raft reorganization, redistribution of membrane-associated signaling receptors, and modulation of ion channel activity, all of which are required for fertilization competence [28].

In contrast, CHO sequestration from the sperm membrane does not necessarily require bicarbonate influx. This mechanism appears particularly relevant in amphibians and aquatic animals, in which sperm function in freshwater or seawater environments containing limited bicarbonate. In *Bufo arenarum*, sperm undergo capacitation-like changes following exposure to egg jelly components that promote CHO removal and activate cyclic adenosine monophosphate-dependent signaling pathways [29]. Similarly, crustacean sperm exposed to factors in the female reproductive tract or to CHO-sequestering molecules exhibit reduced membrane CHO and enhanced PTP, supporting a bicarbonate-independent activation pathway [15, 16]. The present study extends this concept by demonstrating that recombinant PpNPC2 alone is sufficient to induce CHO depletion and activate downstream signaling in crab sperm, providing strong evidence for a bicarbonate-independent capacitation-like pathway in brachyuran species. This mechanism may also represent an adaptation shared by other aquatic animals inhabiting bicarbonate-limited environments. Regardless of whether capacitation-like activation proceeds through bicarbonate-dependent or bicarbonate-independent pathways, CHO sequestration appears to be a pivotal regulatory event. This process requires CHO-binding proteins that can modulate membrane lipid composition. Among these proteins, NPC2 has emerged as one of the most consistent regulators of sperm activation in aquatic animals [14–16].

### NPC2-mediated sterol trafficking is associated with conserved sperm activation signaling

Recent electrophysiological and molecular studies have demonstrated that sterol-dependent membrane remodeling is closely coupled to ion channel regulation, intracellular Ca²⁺ homeostasis, and changes in membrane potential during sperm activation [6, 27, 30, 31]. Together, these findings reinforce the concept that membrane sterol dynamics function as upstream regulators of sperm competence. In the giant freshwater prawn *Macrobrachium rosenbergii*, recombinant NPC2 removes CHO from testicular sperm membranes and promotes the transition to physiologically active sperm accompanied by increased PTP [15, 16]. A comparable response was observed in the present study, in which recombinant PpNPC2 reduced membrane CHO and promoted sperm activation in brachyuran sperm. Similar to mammalian capacitation, the downstream signaling pathway likely involves Ca²⁺ influx and cAMP-protein kinase A activation, culminating in increased PTP [1, 5]. Consistent with this hypothesis, marked enhancement of PTP was observed following PpNPC2 treatment (Figure 6). These findings suggest that similar intracellular signaling pathways also operate during brachyuran sperm activation. Collectively, the functional conservation observed between prawns and crabs indicates that NPC2-mediated sterol trafficking represents an evolutionarily conserved mechanism regulating sperm maturation among aquatic decapods.

### Morphological remodeling accompanies brachyuran sperm capacitation-like activation

In addition to these biochemical changes, brachyuran sperm underwent pronounced morphological remodeling during the capacitation-like process, which differed substantially from that previously described in prawns (Supplementary Figure 4). Tsp exhibited a compact round morphology, whereas Ssp displayed progressive acrosomal protrusion and ultimately acquired the characteristic snowman-like morphology (Figure 5). This morphological transformation is unique to brachyuran sperm [22, 32, 33] and represents either a capacitation-like process or acrosomal reversion, as previously described in *Eriocheir sinensis* [17]. Regardless of its designation, this structural remodeling appears to be closely associated with functional maturation and the acquisition of fertilization competence. Such reorganization is likely coordinated by cytoskeletal remodeling and Ca²⁺-dependent membrane fusion events that facilitate subsequent gamete interactions.

### Evolutionary significance of PpNPC2-mediated sperm activation

From an evolutionary and ecological perspective, externally fertilizing marine decapods, including brachyuran crabs, release their gametes into environments characterized by considerable fluctuations in salinity, temperature, and pollutant exposure [34]. Therefore, sperm activation before release from the male reproductive tract is likely an intrinsic physiological adaptation that ensures fertilization competence prior to external spawning. Consistent with this concept, the present study demonstrated that PpNPC2 is localized in the male reproductive tract, where it functions as an endogenous CHO-sequestering protein that primes sperm during transit through the spermatic duct prior to gamete release. These findings provide new evidence that capacitation-like sperm activation is not restricted to mammals but also occurs in externally fertilizing marine crustaceans through an evolutionarily conserved sterol-mediated mechanism.

### Practical implications, study limitations, and future perspectives

The present findings also have important implications for crustacean reproductive biotechnology. Because PpNPC2 effectively induced capacitation-like changes *in vitro*, it may serve as a valuable tool for controlled sperm activation before cryopreservation and the development of assisted reproductive technologies, including *in vitro* fertilization, in brachyuran species. However, this study has several limitations. Functional fertilization assays and targeted NPC2 knockout or knockdown experiments were not performed; therefore, the direct requirement of PpNPC2 for fertilization remains to be established. Nevertheless, the integrated biochemical, morphological, and signaling evidence presented here substantially extends previous observations in shrimp species and provides the first mechanistic evidence for NPC2-mediated capacitation-like activation in brachyuran sperm. Future studies combining functional genetic approaches with in vivo fertilization experiments will further clarify the physiological significance of NPC2-mediated sperm activation and facilitate its application in crustacean reproductive management.

## CONCLUSION

This study provides the first evidence that NPC2 functions as an endogenous mediator of CHO sequestration during sperm maturation in brachyuran crabs, thereby establishing a previously unrecognized capacitation-like mechanism in *P. pelagicus*. Recombinant PpNPC2 exhibited specific CHO-binding activity and effectively reduced membrane CHO levels in Tsp, accompanied by progressive morphological remodeling from the typical round sperm to the characteristic snowman-like morphology observed in Ssp. These structural changes were closely associated with enhanced PTP, indicating that PpNPC2 not only mediates membrane lipid remodeling but also activates downstream signaling events required for sperm functional maturation. The localization of PpNPC2 in epithelial cells of the spermatic duct, together with its absence in sperm, further supports a physiological role for the male reproductive tract in regulating sperm activation prior to gamete release.

A major strength of this study is the integration of complementary molecular, biochemical, structural, histological, and functional approaches to elucidate the role of NPC2 in brachyuran sperm physiology. Sequence characterization, structural modeling, recombinant protein production, CHO-binding assays, immunohisto-chemistry, lipid profiling, immunofluorescence, Western blotting, and functional induction experiments collectively provide converging evidence supporting NPC2-mediated sperm activation. The concordance between endogenous sperm maturation during spermatic duct transit and the *in vitro* effects induced by recombinant PpNPC2 further strengthens the proposed mechanism.

Collectively, these findings demonstrate that NPC2-mediated CHO sequestration represents a conserved yet previously undescribed mechanism underlying capacitation-like activation in brachyuran sperm. This work broadens the current understanding of sperm maturation beyond mammalian systems, provides new insights into the evolution of reproductive mechanisms in aquatic invertebrates, and establishes a mechanistic foundation for future studies investigating NPC2-regulated signaling pathways, functional fertilization, and the development of reproductive biotechnologies, including sperm cryopreservation and *in vitro* fertilization, in commercially important brachyuran species.

## DATA AVAILABILITY

The supplementary data can be made available from the corresponding author upon request.

## GENERATIVE AI DECLARATION

During the preparation of this manuscript, the authors used Grammarly to improve the text’s language, spelling, and grammar. After using this tool, the authors reviewed and edited the content as needed and take full responsibility for the final framework and content of the published article. No generative AI tools were used to create a draft or contribute to the scholarly ideas or data interpretation of this work.

## AUTHORS’ CONTRIBUTIONS

WW and TS contributed equally to this work as first authors. SA, PS, and WW: Conceptualization. WW, SA, PS, TS, TK, OT, SB, and JS: Visualization, formal analysis, data curation, validation, and writing – original draft preparation, review, and editing. SA, PS, and WW: Supervision. WW, TS, TK, OT, SB, and PS: Investigation. TS, TK, OT, SB, JS, and PS: Methodology. SA and PS: Project administration. All authors have read and approved the final version of the manuscript.
